# Drought-Induced Responses of Nitrogen Metabolism in *Ipomoea batatas*

**DOI:** 10.3390/plants9101341

**Published:** 2020-10-11

**Authors:** Houqiang Xia, Tao Xu, Jing Zhang, Ke Shen, Zongyun Li, Jingran Liu

**Affiliations:** Institute of Integrative Plant Biology, Jiangsu Key Laboratory of Phylogenomics and Comparative Genomics, School of Life Science, Jiangsu Normal University, Xuzhou 221116, China; xhq985410178@163.com (H.X.); taoxu@jsnu.edu.cn (T.X.); zhangjing_85005@163.com (J.Z.); shenke199511@163.com (K.S.)

**Keywords:** *Ipomoea batatas* sweet potato, nitrate uptake, ammonium uptake and accumulation, gene transcription, drought stress

## Abstract

This study investigated the effect of water stress, simulated by the polyethylene glycol (PEG-6000) method, on nitrogen (N) metabolism in leaves and roots of hydroponically grown sweet potato seedlings, Xushu 32 (X32) and Ningzishu 1 (N1). The concentrations of PEG-6000 treatments were 0%, 5% and 10% (m/v). The results showed that the drought-treated plants showed a decline leaf relative water content, and revealed severe growth inhibition, compared with the 0% treatment. Under drought stress, the decline in biomass of the leaf and stem was more noticeable than in root biomass for X32, leading to a higher root to shoot ratio. Drought stress increased the nitrate nitrogen (NO_3_^−^-N) and protein in leaves, but reduced all the activities of N-metabolism enzymes and the transcriptional levels of nitrate reductase (*NR*), glutamine synthetase (*GS*) and glutamate synthase (*GOGAT*); in roots, NO_3_^−^-N and NR had opposite trends. The leaf ammonium nitrogen (NH_4_^+^-N), GS and amino acid had different trends between X32 and N1 under drought stress. Furthermore, the transcriptional level of nitrate transporter genes *NRT1.1* in leaves and roots were upregulated under drought stress, except in N1 roots. In conclusion, NR determined the different response to drought in leaves for X32 and N1, and GS and GOGAT determined the response to drought in roots, respectively.

## 1. Introduction

Sweet potato (*Ipomoea batatas* (L.) Lam.) is a vital grain and forage crop in China. It has strong drought resistance and wide adaptability, and is often used as a drought relief crop [1]; however, during growth and the development of maturity the plants are susceptible to drought [2]. Drought has become a major environmental factor limiting global crop yields in various abiotic stresses [3]. In China, more than 70% of sweet potato is planted in hilly and mountainous areas. In these areas, the annual rainfall of the sweet potato growth season is uneven, and drought is frequent, resulting in the serious decline in the tuberous yield of the sweet potato. Rapid root formation after planting is the basis to ensure the survival rate of sweet potato seedlings and root tuber formation [4]. The sweet potato is very sensitive to soil moisture at the seedling stage. It is suitable for soil moisture to be 60–75% of the maximum water holding capacity in the field for the sweet potato, and irrigation should be carried out when the soil relative moisture content is less than 60% [5], and less than 50% of the soil relative water content (RWC) will directly affect the formation, growth and differentiation of adventitious roots. While at lower soil moisture, more soil resistance restricted growth and tuber development, at the same time less labile N and K were transferred from the soil reducing tuber dry matter production and tuber yield [6].

As one of the three major nutrients, nitrogen (N) greatly contributes to the increased yield of major food crops [7]. Generally, nitrate nitrogen (NO_3_^−^-N) and ammonium nitrogen (NH_4_^+^-N) are mainly taken up in plants, rather than amino acids (AAs) or other N organic forms. Moreover, NO_3_^−^-N is also the regulatory factor in response to N metabolism, thus playing a critical role in regulating carbon absorption, the carbon and N absorption ratio and light cooperation efficiency [8]. NH_4_^+^-N plays a crucial role in the N acquisition regulation by influencing the transcription and/or activity of root NO_3_^−^ transport systems [9]. In sugarcane, drought stress reduced NO_3_^−^ and NH_4_^+^ uptake [8]. Additionally, the nitrate transporter family (*NRT1:1* and *NRT2.1*) may play crucial roles in NH_4_^+^ and NO_3_^−^ uptake in winter wheat (*Triticum aestivum* L.) and *Populus* [10,11]. Nitrate reductase (NR) is a key rate-limiting enzyme that regulates nitrogen metabolism and assimilation and is sensitive to environmental conditions [12]. Glutamine synthetase (GS), key enzyme in the NH_4_^+^ assimilation, is encoded by genes expressed in the cytoplasm or root plastid [13], which regulation is determined by a vast number of biotic and abiotic factors. Under water stress, NH_4_^+^ produced through NR and nitrite reductase-mediated (NiR) reduction of NO_3_^−^ was reduced as well as the activities of GS and glutamate synthase (GOGAT). These results led to ammonium accumulation and toxic effects on plants [14]. Glutamate dehydrogenase (GDH) also plays a major role in the NH_4_^+^ assimilation and is determined by stress factors [13,15]. Under water stress, NR and nitrite reductase, mediated by NO_3_^−^ reduction, was reduced as well as the activities of GS and GOGAT, leading to NH_4_^+^ accumulation and toxic effects on plants [16]. There are also studies demonstrating that drought stress increased NR activity, and produced sufficient nitric oxide, resulting in higher nitric oxide synthase activity in adventitious roots [17], and the activities of GS, GOGAT and GDH was increased for dwarf bamboo [18]. Additionally, in maize, drought stress has also enhanced the activities of hydrolytic enzymes, soluble protein (SP) content, some osmotic regulators containing nitrogen, such as AAs and amides, which can reflect the degree of water stress [19]. Therefore, the decrease of crop yield under soil drought can be partly attributed to the hindrance of N absorption and utilization, and different crops respond differently to drought stress [20,21].

In recent years, previous studies mainly focused on the root morphological structure, the production of reactive oxygen species (ROS) and the relationship between physiological and biochemical indexes such as photosynthetic capacity, endogenous hormones and antioxidant enzyme activity and drought resistance of sweet potato varieties [22,23,24,25,26]. Under drought stress, the net photosynthetic rate, stomatal conductance, water use efficiency, maximum photochemical efficiency of PS II, quantum yield and photochemical quenching coefficient of sweet potato plants were reduced, and the transpiration rate, non-photochemical quenching coefficient and stomatal limit value of sweet potato leaves were increased [27]. Additionally, under drought stress, ABA, proline content was significantly increased, whereas malonaldehyde (MDA) and H_2_O_2_ content was significantly decreased in the transgenic sweet potato plants [3]. Moreover, drought stress significantly reduced the average diameter and root volume [28]. However, few articles on N metabolism in sweet potato have been reported, only one about the changes of ion homeostasis and N metabolism of the sweet potato in response to salt stress [29]. N metabolism in response to drought stress for the sweet potato has, to our knowledge, not been well studied.

In the present study, drought-induced changes of inorganic N, activities of the main N-metabolism enzymes and the related genes transcription in sweet potato seedlings were investigated. The purpose of this study was (1) to evaluate the N metabolism to the overall drought tolerance at the adventitious root stage, (2) to analyze relationship between leaf RWC and N metabolism in sweet potato seedlings and (3) to study the relationship of N transporter genes with NO_3_^−^. The changes induced by drought stress in N metabolism in sweet potato seedlings may be considered to implement water management strategies to cope with the damage caused by drought.

## 2. Results

### 2.1. Leaf RWC and Membrane Stability Index (MSI)

**Figure 1:**
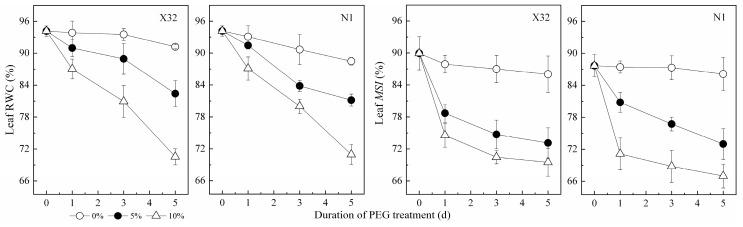
Effects of drought stress on relative water content (RWC) and the membrane stability index (MSI) in sweet potato leaves. X32 and N1 represent Xushu 32 and Ningzishu 1, respectively.

**Figure 2:**
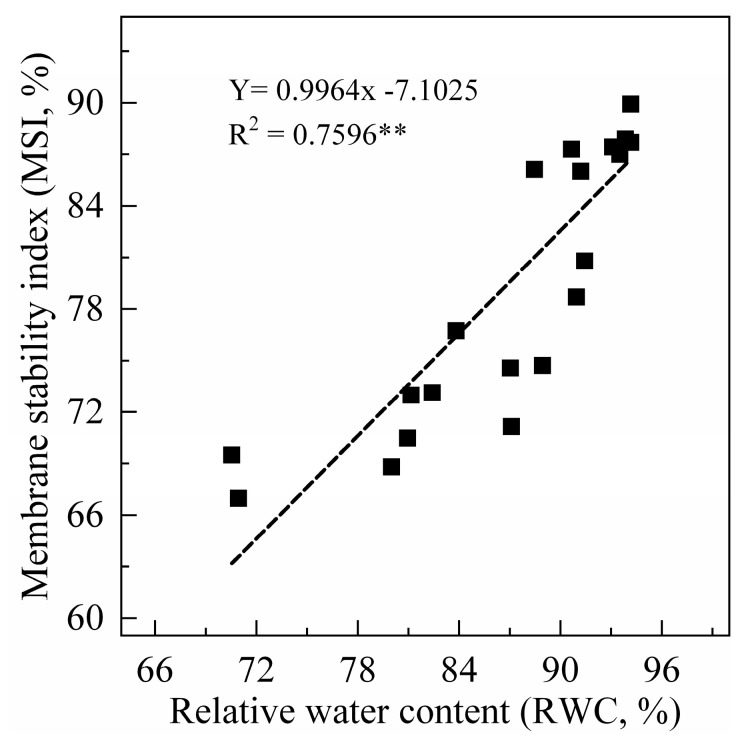
Correlation analysis between relative water content (RWC) and membrane stability index (MSI) in sweet potato leaves. *n = 24,* R^2^_0.05_ = 0.164, and R^2^_0.01_ = 0.265.

Leaf RWC can truly reflect the degree of water deficit in plants under drought stress. In this study, the leaf RWC of sweet potato seedlings showed a decreasing trend after drought stress, and the difference increased with increasing PEG concentration (Figure 1). Leaf membrane stability index (MSI) had a similar trend to leaf RWC. When the drought treatment lasted for five days, compared to the 0% treatment, the leaf RWC and MSI decreased by 8–23% and 15–22% under drought stress, respectively. In addition, leaf RWC was significantly positively correlated with MSI (Figure 2).

### 2.2. Growth Parameters in the Sweet Potato

**Figure 3:**
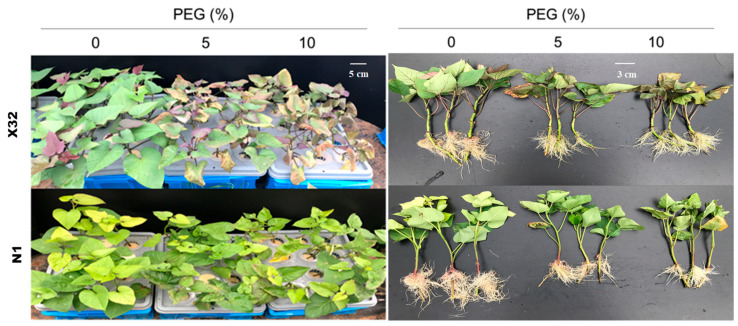
Effect of PEG-induced drought stress on plant growth and development in the sweet potato. Each treatment consisted of five pots, each containing twelve plants. X32 and N1 represent Xushu 32 and Ningzishu 1, respectively.

**Figure 4:**
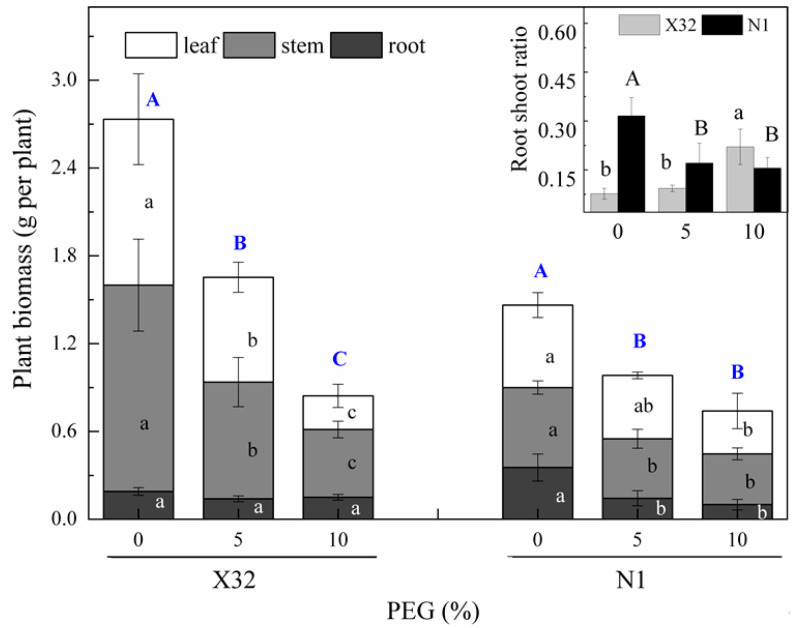
Growth parameters of sweet potato seedlings as affected by the different PEG concentrations. X32 and N1 represent Xushu 32 and Ningzishu 1, respectively. Values followed by the different lowercase letters within the same cultivar in the main part are significantly different (*p* < 0.05) among the three PEG levels for leaf, stem and root biomass, and by the different big letters within the same cultivar in the main part are significantly different for plant biomass. Values followed by different small letters within the same cultivar in the input part are significantly different (*p* < 0.05) among the three PEG levels for X32, and by different capital letters for N1.

After 5 days of the drought treatment, serious growth inhibition was observed in the PEG-treated plants (Figure 3), leading to a decline in biomass compared with the 0% PEG-induced treatment (the controls, Figure 4, main part). Drought stress obviously reduced the stem biomass, leaf biomass and total plant biomass (Figure 4, main part). Compared to Ningzishu 1 (N1), drought stress induced an even higher reduction for the three indices in Xushu 32 (X32). The 5% and 10% PEG-induced drought reduced stem, leaf and total biomass by 44–67%, 37–80% and 40–69% in X32, respectively, and by 26–37%, 23–48% and 33–49% in N1. However, there was an opposite trend for the root biomass.

Moreover, drought had a significant effect on the root to shoot ratio (R/S, Figure 4, inset). With increasing PEG concentration, the R/S significantly increased in X32, while it significantly decreased in N1.

### 2.3. Chlorophyll Content in Leaves

**Figure 5:**
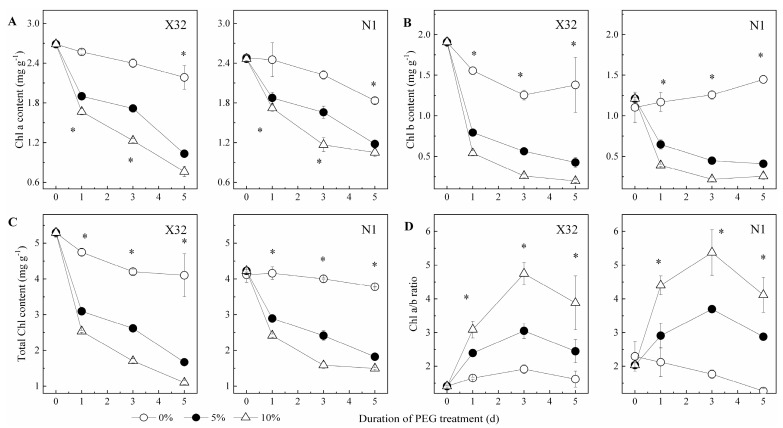
Effects of drought stress on chlorophyll content (**A**: Chl a content; **B**: Chl b content; **C**: Total Chl content; **D**: Chl a/b ratio) in sweet potato leaves. X32 and N1 represent Xushu 32 and Ningzishu 1, respectively. The stages labeled with an asterisk (*) indicate significant differences (*p* < 0.05) among the three PEG levels in the figure.

Drought markedly reduced chlorophyll (Chl) content (Figure 5A–C). As for sweet potato in the 5% and 10% PEG treatment, the content of Chl a was increased by 25–35% for X32 and by 20–29% for N1, respectively. A significant decrease in the content of Chl b (58–90% for X32 and 42–79% for N1) and total Chl (46–80% for X32 and 32–65% for N1) were also observed. Moreover, X32 had higher Chl content than N1.

The Chl a/b ratio increased with the extension of the drought time and the aggravation of the drought degree (Figure 5D). It was thus indicated that Chl a content fell more slowly than Chl b under drought stress. Furthermore, the increased degrees of X32 among different drought treatments were higher than N1.

### 2.4. NO_3_^−^-N and NH_4_^+^-N, AA and SP Content in Leaves and Roots

**Figure 6:**
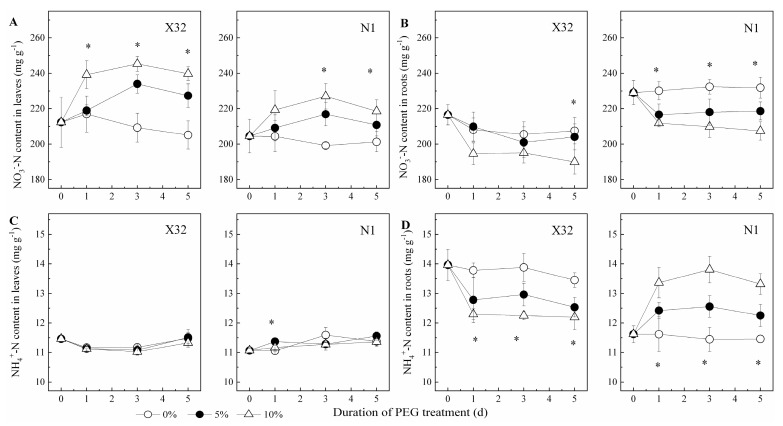
Effects of drought stress on NO_3_^−^-N and NH_4_^+^-N content in leaves (**A**,**C**) and roots (**B**,**D**) of the sweet potato. X32 and N1 represent Xushu 32 and Ningzishu 1, respectively. The stages labeled with an asterisk (*) indicate significant differences (*p* < 0.05) among the three PEG levels in the figure.

**Figure 7:**
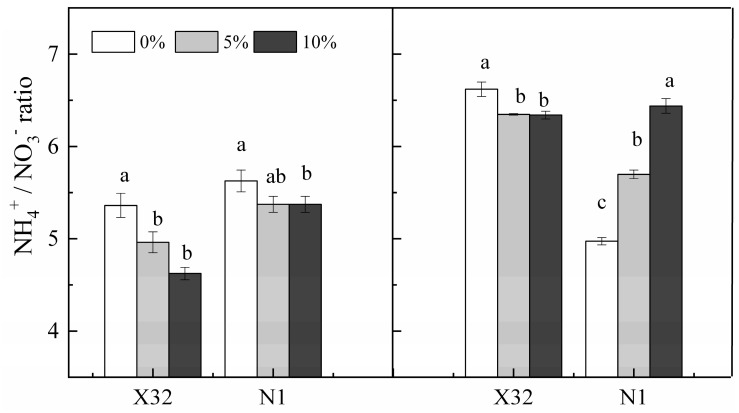
Effects of drought stress on the NH_4_^+^/NO_3_^−^ ratio in leaves and roots of the sweet potato. X32 and N1 represent Xushu 32 and Ningzishu 1, respectively. Different letters in the same cultivar indicate significant differences (*p* < 0.05) among the three PEG levels in the figure.

A sharp NO_3_^−^-N increase was found after PEG-induced drought stress in leaves for the two cultivars (Figure 6A). Contrary to leaves, NO_3_^−^-N content was remarkably reduced by drought stress in roots (Figure 6B). However, NH_4_^+^-N content remained constant in leaves of PEG-treated X32 and N1 (*p* > 0.05, Figure 6C). Drought stress quickly reduced NO_3_^−^-N in X32 roots, whereas an increased NO_3_^−^-N in roots were observed for N1 (Figure 6D). Additionally, drought stress reduced the NH_4_^+^ to NO_3_^−^ ratio in leaves and roots, except in N1 roots with an increasing trend (Figure 7).

**Figure 8:**
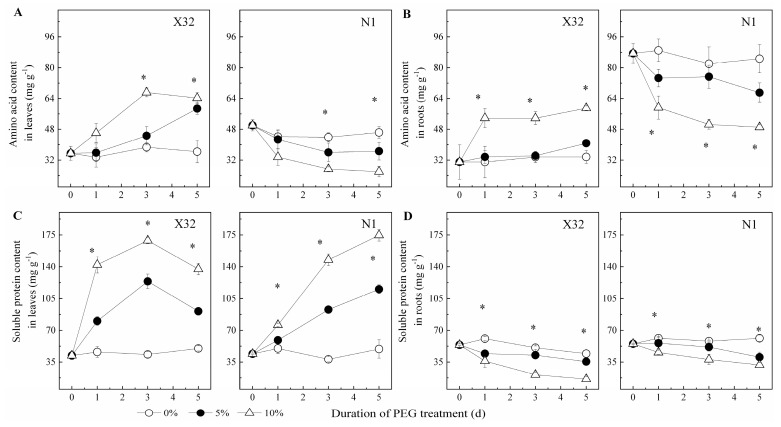
Effects of drought stress on amino acid and soluble protein content in leaves (**A**,**C**) and roots (**B**,**D**) of sweet potato. X32 and N1 represent Xushu 32 and Ningzishu 1, respectively. The stages labeled with an asterisk (*) indicate significant differences (*p* < 0.05) among the three PEG levels in the figure.

Drought stress significantly increased AA in leaves and roots of X32, but an opposite trend was observed upon drought induction in N1 (Figure 8A,B). Drought-stressed plants showed 21–48% higher AA content in leaves and 10–25% higher in roots as compared to 0% PEG-treated X32, whereas a 10–26% reduction in leaves and 11–28% in roots were observed as compared to 0% PEG-treated N1. Leaf SP content was significantly enriched under drought stress (Figure 8C). Contrary to leaves, SP content was remarkably reduced by drought stress in roots (Figure 8D). 

**Figure 9:**
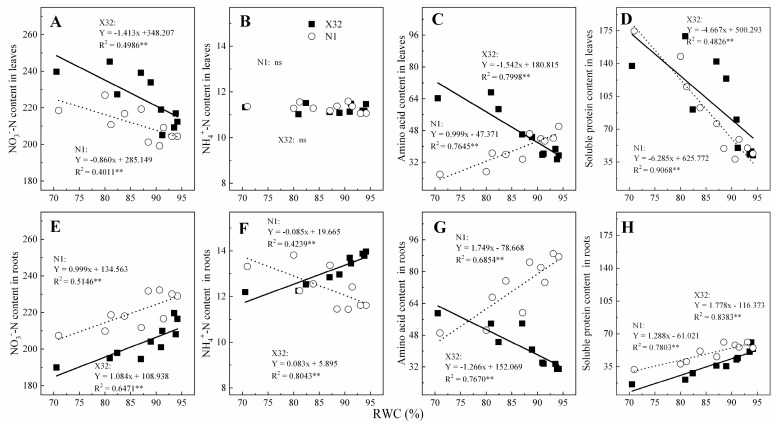
Relationship between NO_3_^−^-N, NH_4_^+^-N, amino acid or the soluble protein content with leaf relative water content in leaves (**A**–**D**) and roots (**E**–**H**) for the two sweet potatoes. The solid and dotted lines represent X32 and N1, respectively.

Furthermore, the relationship of NO_3_^—^N, NH_4_^+^-N, AA and SP with leaf RWC was analyzed (Figure 9). Leaf NO_3_^−^-N was significantly negatively correlated with leaf RWC for both cultivars (Figure 9A), with the slope of the fitted line in X32 being higher than that of N1. However, there was non-significant correlation between leaf NH_4_^+^-N and leaf RWC for the two cultivars (*p* > 0.05, Figure 9B). Root NO_3_^−^-N and NH_4_^+^-N was also significantly linearly correlated with leaf RWC (Figure 9E,F), with the slope of the fitted line in X32 also being higher than that in N1. There was a significant negative correlation between leaf AA for X32, SP for the two sweet potato and leaf RWC (*p* < 0.05, Figure 9C,D), and a significant negative correlation between leaf AA and leaf RWC for N1 (*p* < 0.05). Whereas, the content of AA and SP in roots showed an opposite correlation with a linear response to leaf RWC (*p* < 0.05, Figure 9G,H).

### 2.5. Changes in N Metabolism Enzymes in Leaves and Roots

**Figure 10:**
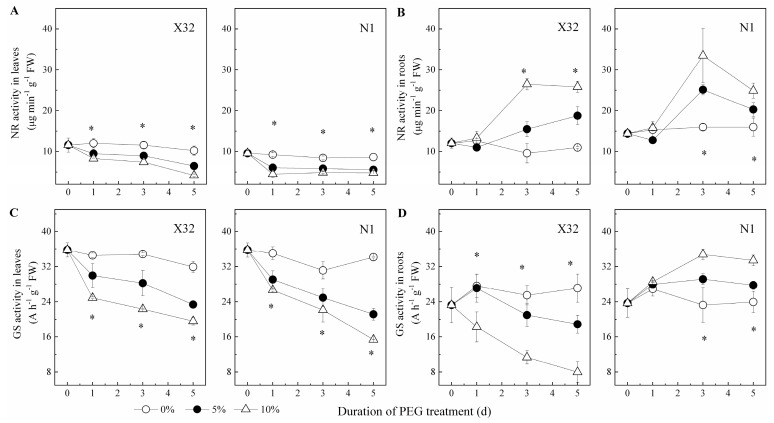
Effects of drought stress on NR activity and GS activity in leaves (**A**,**C**) and roots (**B**,**D**) of the sweet potato. X32 and N1 represent Xushu 32 and Ningzishu 1, respectively. The stages labeled with an asterisk (*) indicate significant differences (*p* < 0.05) among the three PEG levels in the figure.

**Figure 11:**
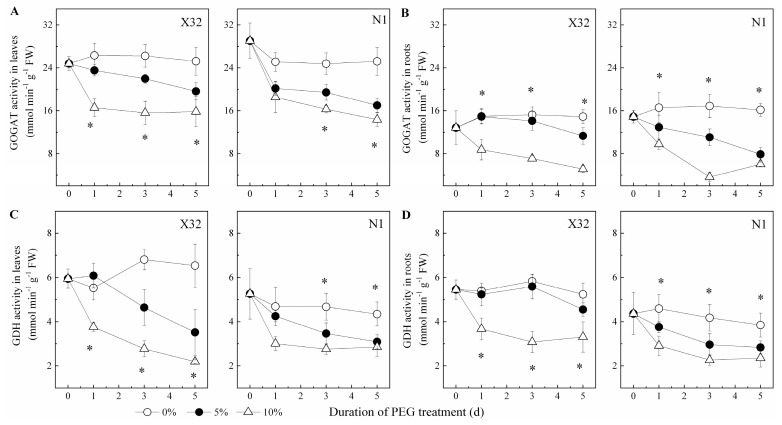
Effects of drought stress on GOGAT activity and GDH activity in leaves (**A**,**C**) and roots (**B**,**D**) of the sweet potato. X32 and N1 represent Xushu 32 and Ningzishu 1, respectively. The stages labeled with an asterisk (*) indicate significant differences (*p* < 0.05) among the three PEG levels in the figure.

NR activity increased in leaves, but showed an opposite trend in roots (Figure 10A,B). After exposure to drought stress, the drought-treated sweet potato showed a significantly lower GS activity in both leaves and roots, except in N1 roots (Figure 10C,D). The activity of GOGAT and GDH was remarkably reduced in roots and leaves of drought-treated plants (Figure 11). In X32 leaves, NR, GS, GOGAT and GDH activities was reduced by 20–31%, 14–25%, 12–29% and 18–25% under 5% and 10% PEG-treated conditions, respectively, as compared to 0% PEG-treated plants. Additionally, N1 leaves showed a higher response to drought in NR and GS. Compared to leaves, cultivar differences had an opposite response in roots. In addition, protease activity varied little among the three PEG treatments in leaves and roots (data not shown).

**Figure 12:**
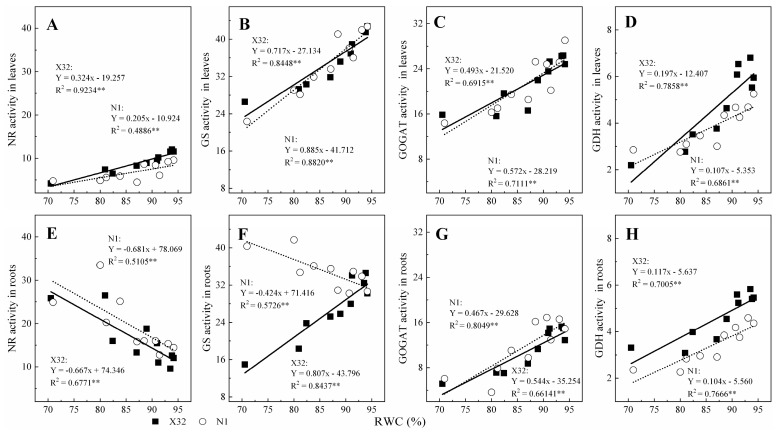
Relationship between NR, GS, GOGAT or GDH activities with the leaf relative water content in leaves (**A**–**D**) and roots (**E**–**H**) for the two sweet potato. The solid and dotted lines represent X32 and N1, respectively.

Furthermore, the relationship between enzyme activity and leaf RWC was investigated (Figure 12). The activity of NR, GS, GOGAT and GDH in leaves and roots could all be related to leaf RWC using a linear equation for both cultivars (R^2^ = 0.4886–0.9234**). There was a positive correlation between NR, GS, GOGAT and GDH with leaf RWC, except NR activity for the two cultivars roots and GS activity for N1 roots, with a negatively correlation with leaf RWC.

### 2.6. Transcript Abundance Related to N Metabolism Exposed to Drought Stress

**Figure 13:**
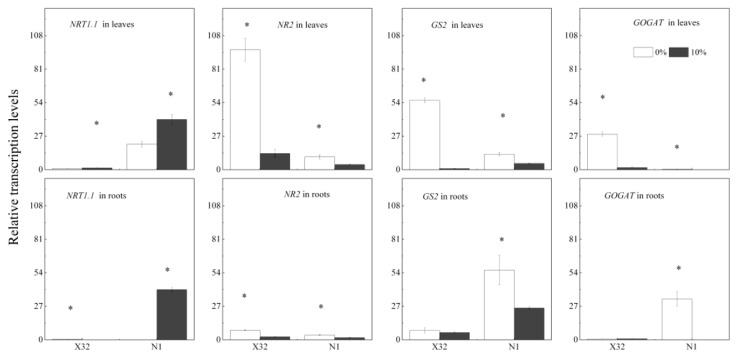
Effects of drought stress on the transcription of N metabolism-related genes in leaves and roots of two sweet potato cultivars. Sweet potato seedlings were subjected to 5 days of drought stress (10% PEG); total RNA was isolated from leaves and roots for real-time PCR analysis. X32 and N1 represent Xushu 32 and Ningzishu 1, respectively. The stages labeled with an asterisk (*) indicate significant differences (*p* < 0.05) between the 0% and 10% PEG levels in the figure.

The transcript level of *NR2*, *GS2* and *GOGAT* significantly reduced in leaves and roots, while genes *GS2* and *GOGAT* in X32 roots were not affected by drought stress. Drought induced a 60–96% decrease in the transcript abundance of *NR2*, *GS2* and *GOGAT* in leaves, and a 23% to 1-fold decrease in the three genes transcript abundance in roots. However, there was an opposite regulation in genes *NRT1* involved in nitrate transport in leaves and roots under drought stress, except in X32 roots (Figure 13).

## 3. Discussion

### 3.1. Changes of Biomass, Chl and Inorganic Nitrogen Exposed to Drought Stress

**Figure 14:**
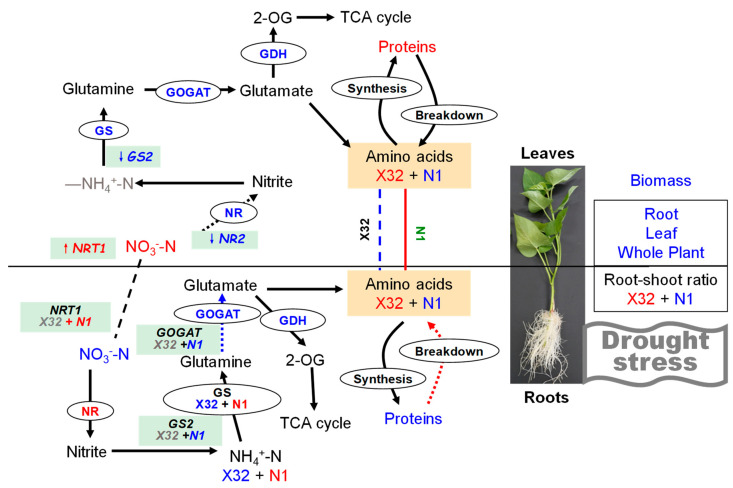
A model of the N regulation in sweet potato seedlings in response to drought stress. Here, drought stress stimulates a N-mediated tandem reaction, which improves the sweet potato drought tolerance. Reducing leaf RWC might alter the expression levels of key regulatory metabolic genes and the activities of N metabolism enzymes, which in turn modulates N accumulation, activates the transcription of NO_3_^−^ transporters to regulate sugar allocation to adapt to environmental stress. Upregulated items under drought stress are marked with red, downregulated items under PEG-induced drought stress are marked with blue, and items that do not change significantly under PEG-induced drought stress are marked with grey.

Drought represents an excellent example of environmental stresses that occur in the field. It is often reported that drought stress leads to the inhibition of plant growth and the changes of physiologic parameters. In the present study, drought stress significantly reduced RWC and MSI, and plant growth was inhibited (Figure 1 and Figure 3). In accordance with previous studies [10,16], PEG-treated plants showed lower stem, root and total biomass (Figure 4). The decrease in leaf RWC and MSI may help the leaves to absorb more water from the solution under hydroponic conditions [30]. However, the effect of drought stress on nitrate reduction and N assimilation in the sweet potato were not fully investigated. In the present study, Chl, NO_3_^−^-N, NH_4_^+^-N and related gene expression involved in N metabolism were obviously influenced by drought stress. In X32, we found that drought inhibited biomass accumulation in leaves and stems more than in roots, resulting in higher R/S in the PEG-treated sweet potato (Figure 1 and Figure 3, as shown in Figure 14), consistent with reports in rice under drought stress [16]. Increased R/S in X32 can be an adaptation trait for crops to increase drought tolerance, because maintaining an effective root system can help to increase water uptake and reduce water loss to cope with drought stress [31]. In our previous study, it was also observed that X32 could make up for part of the loss of yield reduction through its own variety attribute (high yield) under drought stress, even though X32 and N1 belonged to low-resistant varieties [32].

Similar to growth, photosynthetic pigments were damaged in drought-stressed plants, which could directly influence the carbon flux of plants under stress [33]. One possible reason may be that the reduced N metabolism under drought stress led to the decline in Chl content. After all, higher Chl content in plants is an indication of higher leaf N status, which proved that reduced N metabolism may be one reason for lower leaf Chl content in drought-induced sweet potato (Figure 5), as similar results reported in cotton leaves [33]. Under drought stress, the AA content in X32 leaves showed an increasing trend, while that of N1 had an opposite trend. The SP content for X32 and N1 leaves were both increased. Previous studies have reported that exogenous AAs reduced the activity of NO_3_^−^ and NH_4_^+^ absorption and assimilation [8].

### 3.2. Effects of Drought Stress on N Assimilation and Recycling

Generally, plant biomass under stress was reduced, commonly together with the downregulation of N uptake genes [34], such as *NRT1*, one of the genes mediated NO_3_^−^ transport. The transcriptional level of *NRT1* could reflect N absorption and utilization [29]. In the present study, the transcriptional level of *NRT1* was induced by drought stress in sweet potato leaves and roots, except that in X32 roots (Figure 13). It was confirmed that the transcriptional level of *NRT1.1* in a N-efficient cultivar was mainly repressed by drought stress, but its transcriptional level in the N-inefficient cultivar was induced by drought stress [11]. The transcriptional difference of *NRT1* gene in drought-treated plants, to a large extent, reflects the differences in crop species, drought conditions and growth stage.

As a key inorganic N, NO_3_^−^ also plays a critical role in regulating plant growth, osmotic regulation and adaptation to abiotic stress [16]. In the present study, it was observed that drought remarkably declined root NO_3_^−^-N content, but improved leaf NO_3_^−^-N (Figure 6). The variation in NO_3_^−^-N accumulation for leaves and roots was in accord with the trends of root NO_3_^−^-N uptake rate and assimilation. Leaf NO_3_^−^-N plays a critical role in controlling stomatal closing by affecting guard cells depolarization [35]. The reduction of leaf NR activity could be related to a decrease in the photosynthesis rate as a result of stomatal closure, thus inhibiting growth (Table S2). It was confirmed that the reduction of leaf NO_3_^−^-N could utilize the residual energy produced by the photosynthetic apparatus [36]. Hence, the elevated leaf NO_3_^−^-N and lower NR activity revealed that NO_3_^−^ reduction and assimilation were remarkably declined. This phenomenon was unhelpful for avoiding ROS accumulation due to the effects of drought stress, in agreement with previous studies [37]. In addition, reduced NR activity in drought-stressed plants is also largely due to an improved NO_3_^−^ content [15]. Moreover, the mismatched variation in NR activity and NO_3_^−^ content in sweet potato leaves may be attributable to leaf NO_3_^−^ assimilation compartmentalization, as NO_3_^−^ is isolated from cytoplasmic NR, mainly stored in vacuoles [38].

Furthermore, the unaffected NH_4_^+^-N assimilation in sweet potato leaves was probably due to the improved NO_3_^−^ uptake and reduced NO_3_^−^-N pathway in drought-treated leaves (Figure 6C). Nevertheless, the NH_4_^+^ to NO_3_^−^ ratio in X32 and N1 leaves was reduced under drought stress (Figure 7). These results indicate that more of the absorbed NO_3_^−^ was stored under drought stress. Therefore, the resistance of NR activity to drought in sweet potato leaves could be a main factor that ameliorates the sweet potato growth under drought stress, in agreement with *Salicornia europaeae* under abiotic stress conditions, revealing improved NO_3_^−^ uptake [39]. Another reason for increased NO_3_^−^ content in leaves under drought stress was possibly closely related to K; K^+^ is transported with NO_3_^−^ in the xylem from roots to aerial plant parts, as a supplementary cation [10]. In the next studies, the experiments on the interaction between drought and K fertilizer should be arranged.

NR and NiR can convert NO_3_^−^ to NH_4_^+^; thus GS and GOGAT can assimilate glutamine and glutamate to NH_4_^+^. NH_4_^+^-N is necessary for N metabolism in plants, and its efflux maybe depletes NH_4_^+^ in cellular and disrupts the assimilation of NH_4_^+^ [9]. In the present study, both NH_4_^+^-N content, GS and GOGAT activity inX32 roots was reduced under drought stress, whereas NR activity remained increased by drought stress, consistent with NO_3_^−^-N content in roots. These results indicated that the low NH_4_^+^ to NO_3_^−^ ratio in X32 roots was mainly attributed to NH_4_^+^ uptake reductions instead of NO_3_^−^ uptake (Figure 7). However, the prolonged drought time did not decline NH_4_^+^ content and GS activity in N1 roots (Figure 6D and Figure 10D), but drought stress obviously reduced the transcriptional levels of genes *GS2* and *GOGAT* in N1 roots (Figure 13). Indeed, drought stress enhanced the root NH_4_^+^ to NO_3_^−^ ratio in N1. The possibly reason was that NH_4_^+^ has a lower energy requirement compared with NO_3_^−^. Moreover, NH_4_^+^ could stimulate root growth in plants [40].

## 4. Materials and Methods

### 4.1. Plant Materials and Drought Treatment

Sweet potato (*Ipomoea batatas* L., Xushu 32 (X32) and Ningzishu 1 (N1)) seedlings were used in this study. The shoots of X32 were obtained from the laboratory of Zhonghou Tang, Xuzhou Academy of Agricultural Sciences, and the shoots of N1 were obtained from the laboratory of Yizhi Xie in Jiangsu Academy of Agricultural Sciences. Sweet potato seedlings with five leaves were collected from tuberous root (20 days) and to initiate adventitious root growth in 40 cm × 30 cm × 23 cm transfer pots containing 2.5 L quarter-strength modified Hoagland’s solution. The composition of the solution was as following: 1 mM MgSO_4_, 2.5 mM Ca (NO_3_)_2_, 0.2 μM CuSO_4_, 1 μM ZnSO_4_, 0.5 mM (NH_4_)_2_H_2_PO_4_, 1 μM MnSO_4_, 0.1 mM Fe Na EDTA, 20 μM H_3_BO_3_, 5 pM (NH_4_)_6_Mo_7_O_24_ and 2.5 mM K_2_SO_4_. Twelve seedlings were grown in each pot in the greenhouse. The solution was renewed every 48 h. The temperature in the greenhouse ranged from 20 to 25 °C with a photoperiod of 16 h and photosynthetically active radiation of 150 mmol m^−^^2^ s^−^^1^. After seven days, uniform seedlings were chosen to subject to different drought stress treatments produced by adding, in a single step, 0%, 5% and 10% PEG-6000 to the nutrient solution for 5 days. Every pot was repeated, and five pots were grown for each treatment.

At corresponding time points (0, 1, 3 and 5 days after drought stress), three sweet potato seedlings with uniform growth were collected and divided into roots, shoots and leaves. Thereafter, the samples were oven-dried, first at 105 °C for 30 min and then at 65 °C to a constant weight. In the meantime, fine roots or leaves (the 3rd fully expanded main-stem leaf from the top) were collected. In the laboratory, after rinsing with water, the samples were divided into two equal parts. The first half was stored in a refrigerator (−80 °C) for enzyme activities and gene expression analysis, while the second half was dried in an oven follow the above drying method for N assimilation compound analysis. The samples had three biological duplicates.

### 4.2. Leaf RWC and MSI

Leaf fresh sample (FW, 0.3 g) was placed into the distilled water for 4 h, and the weight was recorded as TW. Additionally, then the leaf was oven-dried at 80 °C to a constant dry weight (DW). Leaf RWC was calculated by:

RWC = (FW − DW)/(TW − DW) × 100%(1)

Two leaf fresh samples (0.2 g) were mixed with distilled water (10 mL). One sample was placed at 40 °C for 30 min, and the solution conductivity (C_a_) was determined after removal. The other blade was boiled in water for 10 min, and the conductivity (C_b_) was determined. MSI was calculated by [41]:

MSI = (1 − C_a_/C_b_) × 100% (2)

### 4.3. Chlorophyll, NO_3_*^−^*-N, NH_4_^+^-N and AA Content

Chlorophyll (Chl) was extracted from fresh leaf tissue collected using 50% acetone, mixed in ethanol (25 mL) for 1 day under dark conditions. Chl a, Chl b and Chl (a+b) were then analyzed Enzyme using a microplate reader at 663 and 645 nm [42]. Chl a = 12.21A_663 nm_ − 2.81A_645 nm_, Chl b = 20.13A_645 nm_ − 5.03A_663 nm_ and Chl (a+b) = 20.20A_645 nm_ − 8.05A_663 nm_.

NO_3_^−^-N and NH_4_^+^-N were extracted with distilled water (10 mL) from oven-dried samples (0.2 g) at 100 °C for 1 h to analyze NO_3_^−^-N and NH_4_^+^-N. For NO_3_^−^-N measurement, a 0.1 mL aliquot was taken and added to 5% salicylic acid–sulfuric acid (w/v), the absorption values were measured using a microplate reader at 410 nm [43]. For NH_4_^+^-N measurement, a 0.2 mL aliquot was taken and added to 1 mL reaction solution (2.625 g sodium hydroxide and 1.5 mL hypochlorous acid to a constant volume to 50 mL) and 1 mL chromogenic solution (12.5 mg sodium nitrite and 3 g phenol to constant volume to 50 mL), and then was placed in a water bath at 37 °C for 30 min [44]. The absorption values were measured using a microplate reader at 624 nm. In addition, AAs content was measured using acid ninhydrin [45,46].

### 4.4. Enzyme Extraction and Analysis

NR activity from root and leaf samples was determined according to previous studies [47]. Fresh tissue of leaves and roots (0.15 g) was ground with phosphate buffer (100 mM, pH 7.5, 2 mL). The activity of NR measured the light absorption of residual NO_2_^−^ at 540 nm. The soluble protein (SP) content was analyzed by the G-250 regent using BSA as a standard [48].

GS, GDH and GOGAT were extracted in leaves and roots, as described previously according to previous research [47]. The activity of GS was analyzed using hydroxylamine as a substrate and the production of γ-glutamylhydroxamate was calculated with acidified FeCl_3_ [49]. The activities of GDH and GOGAT were measured by producing NADH oxidation using a microplate reader at 340 nm [47].

### 4.5. Transcriptional Level Analysis of Genes

Total RNA from control and treated roots and leaves were extracted with the DP441-50T RNAprep pure plant kit from Tiangen Biotech (Beijing, China) according to the manufacturer’s instructions. Total RNA (2 mg) was reverse-transcribed using a Prime Script RT reagent cDNA kit (Takara). Afterwards, the synthetic cDNA was used as a template for real-time PCR amplification. The primers of *NRT1.1*, *NR*, *GS* and *GOGAT* were synthesized by Sangon Biotechnology (Shanghai, China). The relative transcriptional levels were calculated using 2^−ΔΔCT^ based on the transcriptional levels of a target gene versus *GAPDH* (a reference gene in sweet potato) [50,51]. Quantitative RT-PCR primers and internal standard primers were shown in Table S1.

### 4.6. Statistical Analysis

Data were shown as average value (±SE), and were analyzed using Origin 2018 and SPSS 23.0 with at least three replications. ANOVA was used to compare the drought response with different letters and/or * via the least significant difference (*LSD*) test (*p* < 0.05).

## 5. Conclusions

Based on these results, a scheme summarizing the effects of drought on primary metabolism in leaves and roots of the sweet potato was proposed (Figure 14). In summary, leaf RWC of sweet potato seedlings showed a decreasing trend after drought stress. The drought-treated plants revealed severe growth inhibition, compared with the 0% PEG-treated plants. The unaffected NH_4_^+^-N assimilation in sweet potato leaves was probably due to the improved NO_3_^−^ uptake and reduced NO_3_^−^-N pathway in drought-treated leaves, mainly determined by NR activity. Both NH_4_^+^-N content, GS and GOGAT activity in X32 roots was reduced under drought stress, whereas NR activity remained increased by drought stress, consistent with NO_3_^−^-N content in roots. The low NH_4_^+^ to NO_3_^−^ ratio in X32 roots was mainly attributed to NH_4_^+^ uptake reductions instead of NO_3_^−^ uptake. However, prolonged drought time did not decline NH_4_^+^ content and GS activity in N1 roots, but drought stress obviously reduced the transcriptional levels of genes *GS2* and *GOGAT* in N1 roots. Indeed, drought stress enhanced root NH_4_^+^ to NO_3_^−^ ratio in N1. Therefore, GS and GOGAT determined the response to drought in roots.

## Supplementary Materials

The following are available online at https://www.mdpi.com/2223-7747/9/10/1341/s1, Table S1: Specific primers for gene amplification. Table S2: Changes of *Pn* and *gs* in sweet potato leaves at one day after PEG-drought stress.
