# Supplementary material for: Drought-Induced Responses of Nitrogen Metabolism in Ipomoea batatas
Source: Plants (Basel). 2020 Oct 11;9(10):1341. doi: 10.3390/plants9101341 (PMC7600920; doi:10.3390/plants9101341)
Supplement: Supplementary file 1 [file plants-09-01341-s001.pdf]

plants

Table S1. Specific primers for gene amplification.

Enzyme/Protein	Target gene	Forward primer (5′→3′)	Reverse primer (5′→3′)
Nitrate transporter 1.1	*NRT1.1*	GGTTGAGGCTTGTGAGAGGTT	GTTATTGGCGGCGTTAGCATTC
Nitrate reductase	*NR2*	GGAATAACGCCGATCTACCAAG	GAATATCCTCCTCCGTCCGATT
Glutamate synthase [NADH]	*NADH-GOGAT*	CAAGGACTTACGAGGTGCTAAC	CGGCTGTCTGTCTCCATGT
Glutamine synthetase	*GS2*	GCCTATCCCAACCAACAAGAG	GCTCCAATGCCACAGTAGTATG
Glyceraldehyde-3-phosphate dehydrogenase	*GAPDH*	ATACTGTGCACGGACAATGG	TCAGCCCATGGAATCTCTTC

Table S2. Changes of *Pn* and *gs* in sweet potato leaves at one day after PEG-drought stress.

PEG/%	*Pn* (mmol CO_2 m^{-2} s^{-1})		*gs* (mol H_2O m^{-2} s^{-1})	
	X32[1]	N1	X32	N1
0	11.45±0.88 a[2]	11.56±1.11 a	0.40±0.013 a	0.34±0.015 a
5	4.75±0.42 b	4.54±0.16 b	0.27±0.004 b	0.20±0.007 b
10	1.71±0.14 c	1.94±0.25 c	0.14±0.017 c	0.09±0.003 c

[1] X32 and N1 represent Xushu 32 and Ningzishu 1, respectively. [2]Values followed by the different letters within the same cultivar in the same column are significantly different at $p = 0.05$ probability level among the three PEG levels.
